# Markerless tumor tracking for lung SBRT with the Radixact Synchrony system: Initial experience and short‐term clinical outcomes

**DOI:** 10.1002/acm2.70362

**Published:** 2025-11-21

**Authors:** Evren Ozan Goksel, Zeynep Ozen, Seckin Gunduz, Artunc Ture, Halil Kucucuk, Alptekin Arifoglu, Evrim Tezcanli, Ufuk Abacioglu, Meric Sengoz

**Affiliations:** ^1^ Vocational School of Health Services, Radiotherapy Program Acibadem MAA University Istanbul Turkey; ^2^ Department of Radiation Oncology Acibadem Altunizade Hospital Istanbul Turkey; ^3^ Department of Radiation Oncology Istanbul Onkoloji Hospital Istanbul Turkey; ^4^ Department Of Medical Sciences School Of Medicine, Radiation Oncology Acibadem MAA University Istanbul Turkey

**Keywords:** lung SBRT, markerless tumor tracking, motion management, radixact synchrony

## Abstract

**Purpose:**

To report the initial clinical experience with the radixact synchrony lung with respiratory (LWR) technique in stereotactic body radiotherapy (SBRT) for lung tumors, focusing on feasibility, dosimetric performance, and early clinical outcomes using both standard and extended fractionation regimens.

**Methods:**

Twenty patients with primary or metastatic lung tumors were treated using the Synchrony LWR technique. Patients were selected based on respiratory stability and favorable imaging characteristics. All patients underwent a Synchrony simulation, which included tumor tracking volume (TTV) definition and kV radiograph acquisition to assess their suitability for treatment with this technique. Treatment plans were generated using Precision TPS with VoloUltra. Plan verification, including gamma analysis and point dose measurements, was performed using the PTW Octavius system and dynamic motion QA. Synchrony model parameters, treatment times, and intrafraction interruptions were analyzed. Clinical follow‐up included imaging and toxicity assessment.

**Results:**

Treatment was successfully delivered in all patients with median beam‐on and treatment durations of 9.1 and 14.5 min, respectively. The median number of intrafraction interruptions was 1.95, and none of the fractions were canceled due to prolonged delivery. The mean kV imaging dose per fraction was 0.90 cGy. Dosimetric QA under motion showed gamma passing rates >93% and < 2% deviation in point dose. At a median follow‐up of 9 months, the local control rate was 100%, with no grade ≥2 toxicities observed.

**Conclusions:**

The Radixact Synchrony LWR technique enables efficient and non‐invasive respiratory motion management in lung SBRT. This early experience demonstrates its feasibility with clinically acceptable delivery accuracy and promising short‐term clinical outcomes.

## 1 INTRODUCTION

Stereotactic body radiotherapy (SBRT) is an established treatment for medically inoperable primary or metastatic thoracic tumors, offering high local control and low toxicity when accurately delivered.^1^, ^2^, ^3^, ^4^, ^5^, ^6^ However, respiratory‐induced tumor motion can compromise treatment efficacy, particularly in centrally located tumors or those adjacent to critical structures.

To manage this challenge, motion management strategies such as four‐dimensional CT (4DCT)‐based internal target volume (ITV) approaches are commonly employed. Although effective, ITV‐based planning increases the irradiated volume of normal tissue, potentially raising toxicity risks.^7^, ^8^, ^9^, ^10^, ^11^ Other strategies—such as respiratory gating, breath‐hold techniques, and abdominal compression—also have limitations. These include prolonged treatment times, poor reproducibility, or infeasibility in patients with impaired lung function.^12^, ^13^, ^14^, ^15^, ^16^ Furthermore, systems relying on external surrogates may not accurately reflect internal tumor motion, resulting in residual uncertainties.

The radixact synchrony lung with respiratory (LWR) technique (Radixact, the next‐generation TomoTherapy System; Accuray Incorporated, Sunnyvale, CA) offers a real‐time, markerless motion management solution by correlating LED‐based external respiratory signals with internal tumor positions captured via kV radiographs acquired at various gantry angles. A predictive model continuously adjusts jaw and MLC positions in real time during free breathing, facilitating effective compensation for respiratory‐induced tumor motion.^17^

In this study, we report our initial clinical experience with the Synchrony LWR system in the treatment of lung tumors, using stereotactic radiotherapy regimens delivered in both standard (≤5 fractions) and extended (up to 10 fractions) schedules. We detail the patient selection criteria, treatment planning, and delivery parameters specific to the LWR technique, and present short‐term clinical outcomes in a cohort of first 20 patients treated at our institution.

## 2 MATERIAL AND METHODS

This study was approved by the institutional review board. Informed consent was obtained from all patients included in the study.

### 2.1 Patient selection

Patients were selected for Synchrony LWR simulation based on specific clinical and technical criteria:^1^ inability to tolerate DIBH,^2^ presence of large respiratory‐induced tumor motion that would otherwise require excessive ITV margins, and^3^ contraindications to MR‐guided radiotherapy such as MR‐incompatible implants (e.g., pacemakers). Additional inclusion criteria included^4^ tumor size between 1 and 8 cm in maximum diameter,^5^ craniocaudal tumor motion less than ± 20 mm.

Ideal candidates were those with island‐type tumors (completely surrounded by aerated lung parenchyma) and regular respiratory motion. Tumors adjacent to the mediastinum or chest wall were also considered when their size, density, and motion characteristics permitted reliable tracking.^17^

Exclusion criteria included^1^ highly irregular respiratory motion,^2^ poor patient cooperation or poor performance status that limited the ability to tolerate extended treatment sessions.

### 2.2 Pretreatment evaluation using Synchrony simulation

The initial evaluation was performed by a medical physicist and a radiation oncologist using recently acquired PET‐CT images. For preliminary assessment, a gross tumor volume (GTV) was delineated, and an LWR simulation plan was created based on the prescribed dose and fractionation scheme using the accuray precision treatment planning system (TPS) (Accuray, Sunnyvale, CA, USA, version 3.3.1.3 [2]). The tumor tracking volume (TTV), which serves as the structure tracked by the system, was derived from the denser solid portion of the tumor and shaped to approximate a more spherical geometry, intentionally excluding finger‐like extensions to enhance visibility on digitally reconstructed radiographs (DRRs) and kV radiographs.^17^ This structure was delineated using the predefined soft tissue window settings (width = 0.167, level = 1.0137) available in the TPS. Six patient‐specific gantry angles were selected for kV radiograph acquisition based on anatomical considerations and expected target visibility. Patients were instructed to maintain regular respiration during simulation; audio coaching was used when necessary to stabilize the breathing pattern.

Of the 22 patients simulated with the LWR technique, 20 proceeded to treatment. In two cases with tumors abutting the diaphragm, the system failed to establish a reliable correlation model due to suboptimal target visibility, and these patients were treated using the ITV‐based method.

### 2.3 Patient setup and simulation for treatment with synchrony

Following pretreatment evaluation using Synchrony simulation, all patients underwent both four‐dimensional computed tomography (4DCT) scans encompassing 10 respiratory phases and an MV‐BHCT scan using the Siemens SOMATOM go.Up scanner integrated with the Varian respiratory gating system. Seventeen patients were positioned with arms up using a wingboard, while three were positioned with arms at their sides due to clinical limitations. A knee wedge was used for comfort.

An MV‐BHCT scan is acquired while the patient holds their breath at the midpoint of the normal tidal breathing cycle. Unlike breath‐hold imaging performed at full inspiration or expiration, this approach provides an anatomically more representative dataset, as it reflects the time‐averaged position of the tumor throughout the respiratory cycle. Moreover, since tumor motion is relatively minimized during breath‐hold, the tumor boundaries appear more clearly defined, enabling more accurate target delineation. Planning CTs were acquired with a slice thickness of ≤3 mm, as recommended in the UK SABR Consortium 2022 guidelines.^18^

MV‐BHCT, which reduces motion blur due to minimal tumor movement, was initially used in the first four patients. However, since pre‐treatment kVCT was obtained during free breathing, registration was challenging. Therefore, for subsequent patients, either the 4DCT phase in which the tumor position corresponded to approximately the mid‐phase of the respiratory cycle (e.g., phase 30, 40, or 50), or the average intensity projection (AIP CT) images reconstructed from the 4DCT dataset, were considered for use as the planning CT image.^19^

In cases where none of the 4DCT phases provided sufficient soft tissue contrast for clear visualization of the TTV, the MV‐BHCT was again selected as the primary dataset for treatment planning.

After the appropriate CT dataset was selected for treatment planning, the GTV was delineated on the selected images. A 3 mm isotropic margin was applied to define the planning target volume (PTV), and no clinical target volume was defined. The TTV was independently contoured using predefined soft tissue window settings to enhance contrast for radiographic visualization.

### 2.4 Treatment planning characteristics

Treatment plans were created using the Accuray Precision TPS with the VoloUltra optimization algorithm. A 2.5 cm field width was selected, and the modulation factor was automatically determined by the system. The Radixact Synchrony system allows for the acquisition of 2–6 kV radiographs per gantry rotation. Since the gantry period directly affects imaging frequency, pitch values were adjusted during treatment planning to achieve an optimal gantry period (less than 20 s) that would enable the acquisition of six kV radiographs per rotation.^20^, ^21^

The planning objectives aimed for ≥98% of the PTV to receive ≥98% of the prescribed dose, while limiting D2% to < 130% of the prescribed dose. High‐dose regions (> 100%) were confined within the GTV. An isotoxic planning strategy was adopted to limit the dose delivered to organs at risk. Therefore, in certain clinical situations, compromises in PTV coverage were made to meet the tolerance dose constraints of critical structures. The 2022 UK SABR Consortium guidelines were followed to evaluate dose conformity, dose gradient, and organ‐at‐risk constraints.^18^

For ultra‐central tumors or reirradiation cases, a 10‐fraction schedule was preferred over standard short‐course SBRT to improve OAR sparing and reduce toxicity risk. These extended regimens maintained SBRT‐level precision, steep dose gradients, and effective protection of adjacent normal tissues.

### 2.5 Synchrony parameter optimization

At the treatment planning stage, acquisition of kV radiographs from six gantry angles was prescribed for all patients, with angle selection tailored to individual anatomy. During the first treatment fraction, both the number and selection of imaging angles were refined based on tracking performance and anticipated target visibility. Gantry angles with poor localization were adjusted or deactivated, ensuring at least four active angles to maintain model stability.

To evaluate tumor motion in three dimensions, center of mass shifts of the GTV across all 10 respiratory phases were assessed using the DICOM Statistics tool in Eclipse (version 16, Varian Medical Systems). Simultaneously, respiratory traces recorded during 4DCT acquisition provided data on respiratory rate and external surrogate amplitude.

The same system parameters were applied during both pretreatment evaluation simulation and treatment. The potential difference threshold and measured delta were both set to 3 mm; the target offset threshold was 30.0 mm. The ‘target outside jaw range’ limit was restricted to a maximum of 10% of treatment time. A 20 mm tracking range and 10‐s auto‐pause delay were used. The sensitivity level was set to medium, and adaptive modeling was enabled.

Prior to each treatment fraction, kVCT imaging was performed to verify patient setup and target localization accuracy.

To promote consistent respiratory motion and minimize intrafractional interruptions, all radiation therapists received dedicated in‐house training in audio coaching techniques to help patients maintain stable breathing during beam delivery.

In case of beam‐hold events due to irregular breathing or tracking errors, the root cause was systematically evaluated. If the interruption was due to respiratory irregularity, the patient was re‐coached using verbal cues, and a new motion model was established. If the system failed to identify the target on the radiographs, imaging parameters such as projection angles or kV mAs values were adjusted to improve target visibility. When necessary, a new kVCT scan was acquired to correct potential setup deviations before reinitiating treatment.

System‐reported data including Synchrony simulation duration, number of acquired kV radiographs, and corresponding patient dose were collected along with treatment parameters such as beam on time, total treatment duration, number of radiographs, and kV imaging dose per fraction.

### 2.6 Patient‐specific plan verification

As part of clinical routine, all treatment plans were first verified using 3D gamma analysis with the PTW Octavius phantom and PTW1500 detector. To incorporate respiratory motion effects, additional QA was performed on a dynamic motion platform (CIRS 008PL) using a Cheese phantom with a high‐density TTV insert.^22^ Dose delivery accuracy under motion was evaluated using absolute point dose measurements and gamma analysis. For point dose verification, the A1SL ionization chamber (Standard Imaging, Inc., Middleton, WI, USA) was used, which has a sensitive (active) volume of 0.053 cm^3^.^23^ For gamma analysis, the PTW 1500 detector was employed. This detector features a checkerboard layout with 5 mm row spacing and a 7.1 mm diagonal nearest‐neighbor distance. It contains 1405 ionization chambers, which can be increased to 2809 effective sampling points by merging two measurements acquired with a 5 mm lateral or longitudinal shift. In our study, this merging technique was applied in all patient‐specific plan verifications. Gamma evaluation was performed based on the AAPM TG‐218 recommendations, using a global 3%/2 mm criterion with a 10% dose threshold.^24^ Previous studies have shown that merged PTW 1500 data yield results comparable to those of the PTW 1000SRS detector and EBT3 film, even under stricter 1%/1 mm criteria.^25^, ^26^

### 2.7 Patient followup

Patient follow‐up was conducted with CT or PET‐CT imaging according to clinical conditions every 3 months. Radiation‐related toxicity was assessed using the common terminology criteria for adverse events (CTCAE), version 5.0.^27^

### 2.8 Declaration of generative AI and AI‐assisted technologies in the writing process

During the preparation of this work, the author(s) used ChatGPT (version GPT‐4o, OpenAI) to assist with language editing, clarity improvement, and formatting of scientific content in English. After using this tool, the author(s) reviewed and edited the content as needed and take(s) full responsibility for the content of the publication

## 3 RESULTS

### 3.1 Patient and target characteristics

The first 20 consecutive patients treated with the Synchrony LWR technique were included in this study. The median patient age was 72 years (range: 59–88 years). Patient characteristics, including gender, primary diagnosis, and treatment intent, are summarized in Table 1. Target volume characteristics, including central versus peripheral tumor location, are summarized in Table 2. The median GTV and PTV were 6.8 cc (range: 0.60–54.4 cc) and 15.3 cc (range: 2.7–100.2 cc), respectively.

**TABLE 1:** Patient demographics, clinical characteristics, and treatment‐related parameters including prescribed dose, beam‐on time, and kV radiograph acquisition details

PATIENT NUMBER	GENDER	DIAGNOSIS	RT OBJECTIVES	FRACTION NUMBER	FRACTION DOSE (Gy)	BEAM‐ON TIME (MIN)	MEAN NUMBER OF KV RADIOGRAPHS	MEAN DOSE FROM Kv RADIOGRAPHS (cGy)
1	M	Thyroid Met	Oligometastasis	3	16.0	9.92	166	0.6
2	M	NSCLC	Definitive	8	7.0	10.02	272	1.0
3	F	NSCLC	Oligometastasis	10	5.0	9.75	439	2.5
4	M	NSCLC	Re‐RT	5	11.0	9.49	60	0.2
5	M	Colorectal Met	Oligometastasis	5	11.0	10.17	193	0.7
6	F	Colorectal	Oligometastasis	8	5.0	5.74	171	0.6
7	M	NSCLC	Re‐RT	5	11.0	13.31	169	0.6
8	M	NSCLC	Oligometastasis	5	10.0	6.96	173	0.6
9	M	SCLC	Oligoresidual	5	10.0	4.81	130	0.5
10	M	RCC Met	Oligometastasis	5	10.0	6.67	236	1.4
11	M	Larynx Met	Oligometastasis	5	10.0	8.49	256	1.5
12	M	NSCLC	Oligometastasis	8	7.0	9.62	290	1.0
13	M	NSCLC	Oligoresidual	10	5.0	7.46	147	0.5
14	M	NSCLC	Definitive	10	5.0	8.97	266	1.0
15	F	NSCLC	Oligoresidual	8	5.0	8.02	188	1.0
16	M	NSCLC	Oligoresidual	8	7.5	5.67	175	0.6
17	F	NSCLC	Definitive	8	7.5	8.02	235	0.8
18	M	NSCLC	Definitive	5	10.0	4.44	190	1.0
19	M	NSCLC	Definitive	10	4.5	8.09	230	1.0
20	F	Breast Met	Oligometastasis	5	8.0	5.19	89	0.3

Abbreviations: F, female; M, male; NSCLC, non‐small cell lung cancer; RCC, renal cell carcinoma, Met, metastasis; Re‐RT, re‐irradiation; SCLC, small cell lung cancer.

**TABLE 2:** Tumor and target volumes characteristics

PARAMETER	MEDIAN	RANGE	*n*
GROSS TUMOR DIAMETER	29 mm	10 – 63 mm	–
GROSS TUMOR VOLUME	6.8 cc	0.6 – 54.4 cc	–
TRACKING TARGET VOLUME	6.8 cc	0.2 – 51.1 cc	
TRACKING TARGET DENSITY	−98.8 HU	−415 – 42 HU	–
PLANNING TARGET VOLUME	15.3 cc	2.7 – 100.2 cc	–
LOCATION			
CENTRAL			9
PERIPHERAL			11

### 3.2 Imaging and treatment planning

MV‐BHCT images were utilized for treatment planning in nine patients, AIP‐CT in three patients, and one of the 4DCT phases corresponding to the mid‐respiratory cycle in eight patients. Specifically, the 30% phase was selected in three patients, the 40% phase in two patients, and the 50% phase in three patients. Evaluation of treatment planning parameters revealed a median pitch value of 0.08 (range 0.05 to 0.13) and a median modulation factor of 1.1 (range 1.0 to 2.5).

### 3.3 Dosimetric verification

The dosimetric accuracy of treatment delivery under simulated respiratory motion was comprehensively analyzed in a separate study.^22^ According to the findings, both gamma analysis and absolute point dose measurements demonstrated strong agreement between planned and delivered doses in the presence of respiratory motion. The median gamma passing rates were comparable for deliveries with and without respiratory motion, measured as 95.1% (range: 90.9%–100.0%) and 93.8% (range: 90.0%–100.0%), respectively. The maximum deviation observed in absolute point dose measurements was less than 2%.

### 3.4 Respiratory metrics and imaging parameters

When the data recorded after the Synchrony simulation were retrospectively evaluated from the ‘treatment report’ section, the median simulation time was found to be 5.27 min (range: 2.25–12.70 min), the median number of acquired kV radiographs was 39 (range: 13–74), and the median additional dose to the patient due to imaging was 0.17 cGy (range: 0.07–0.31 cGy).

The median prescribed dose was 50 Gy delivered in 5 fractions, with a range of 40–60 Gy administered over 3 to 10 fractions (Table1). The mean kV radiographic dose for the group of 20 patients was found to be 0.90 cGy, indicating that, on average, the dose from kV radiographs contributed only 0.11% of the fraction dose.

Table 3 summarizes patient‐specific respiratory pattern characteristics along with the number of intrafractional treatment interruptions observed during delivery. The median respiratory rate was 18 breaths per min (range: 10–25), and the median amplitude of surface motion was 10 mm (range: 6–22 mm). Tumor motion amplitudes were measured as 8 mm (range: 3–15 mm) in the longitudinal, 1.43 mm (range: 0–3.7 mm) in the lateral, and 3.2 mm (range: 0.3–8.8 mm) in the vertical directions. In Patient 11, despite the large LED motion amplitude, tumor motion remained minimal, likely because of its position in the right upper lobe, where respiratory movement is generally less pronounced.^28^

**TABLE 3:** Descriptive data on respiratory metrics, tumor motion, and treatment interruptions

PATIENT NUMBER	(LEDS) MOTION AMPLITUDE (MM)	RESPIRATORY RATE PER MINUTE	SUP‐INF MOTION AMPLITUDE OF THE TUMOR (MM)	ANT‐POST MOTION AMPLITUDE OF THE TUMOR (MM)	LATERAL MOTION AMPLITUDE OF THE TUMOR (MM)	MEAN NUMBER OF TREATMENT INTERRUPTION
1	6.0	20	6.0	1	0.3	1.6
2	9.0	18	10.0	3	1.9	7.3
3	14.0	15	15.0	8.6	3.5	9.5
4	5.0	25	10.0	2.6	1.7	4.0
5	7.0	16	5.0	0.6	0	4.4
6	10.0	20	5.0	0.6	0.1	0.5
7	10.0	19	8.0	1.4	1	0.4
8	10.0	15	16.0	8.7	3.6	0.2
9	8.0	12	5.0	0.4	0.1	0.2
10	7.0	18	3.0	0.3	0	1.2
11	22.0	10	7.0	0.9	0.2	2.6
12	16.0	14	10.0	2.8	1.7	0.3
13	6.0	19	5.0	0.6	0	5.2
14	10.0	20	6.0	0.7	0.1	0.4
15	8.0	21	15.0	8.5	3.4	10.6
16	9.0	12	7.0	1	0.2	0
17	9.0	13	12.0	3.2	2	3.8
18	14.0	10	18.0	8.8	3.7	10.6
19	8.0	23	15.0	8.6	3.3	2.3
20	7.0	14	10.0	2.5	1.8	1.0

### 3.5 Treatment efficiency

The median treatment duration was found to be 14.53 min (range: 6.65–41.58 min). However, after excluding one outlier case, the median treatment duration slightly decreased to 14.33 min, with a narrower range of 6.65–28.90 min. The median percentage increase in treatment duration compared to beam‐on time was calculated as 67.7% (range: 28.0%–531.2%) with the use of the Synchrony technique. However, after exclusion of outlier values, the adjusted median increase was 61.4%, with a range of 28.0%–204.5%.

The number of automatic treatment interruptions had a median of 1.95 (range: 0–10.6). Importantly, none of the Synchrony treatment fractions were canceled due to excessive pauses or prolonged treatment times beyond the 60‐min scheduled slot.

### 3.6 Early clinical outcomes and representative cases

The median follow‐up duration for this patient cohort was 9 months (range: 6–12 months), and the local control rate was 100%. No acute radiation‐related toxicities were observed during treatment. However, radiation‐induced grade 1 pulmonary changes were identified in three patients on follow‐up CT scans.

**FIGURE 1:**
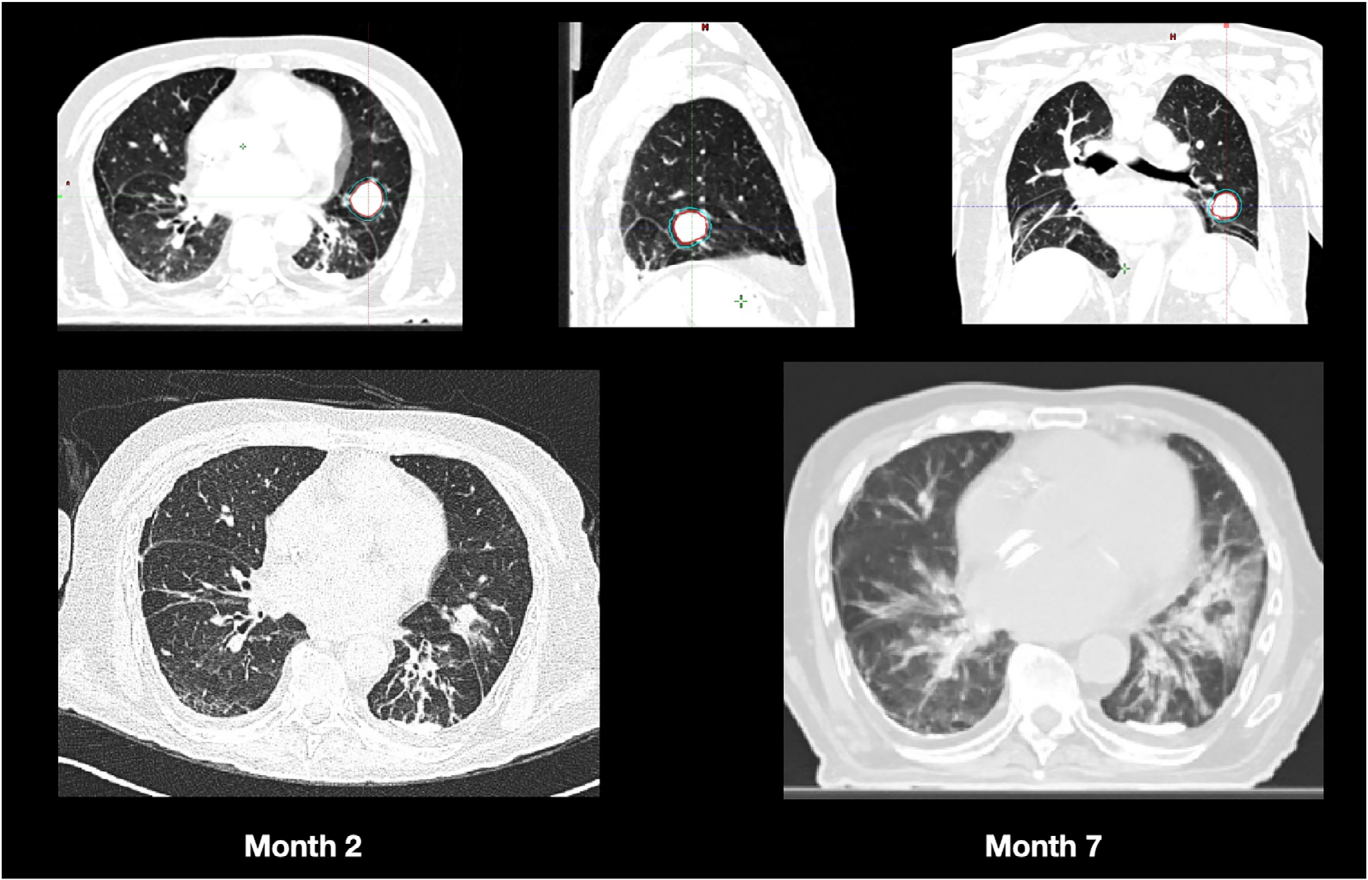
MV‐BHCT planning and follow‐up CT images of a 78‐year‐old male with metastatic colon adenocarcinoma treated for oligometastasis using the Synchrony LWR technique (GTV: 8.2 cc; PTV: 16 cc; 55 Gy/5 fractions).

Figure 1 presents the treatment planning MV‐BHCT image and follow‐up CT images of a representative 78‐year‐old male patient. This patient, diagnosed with metastatic colon adenocarcinoma, underwent treatment using the Synchrony LWR technique for oligometastasis. The tumor diameter was measured as 25 mm, with a GTV of 8.2 cc and a PTV of 16 cc. A total dose of 55 Gy was prescribed in five fractions. The patient received treatment in the arms‐down position on an every‐other‐day schedule. At the 7‐month follow‐up, CT imaging revealed complete response with asymptomatic radiation‐induced changes in the lung parenchyma.

**FIGURE 2:**
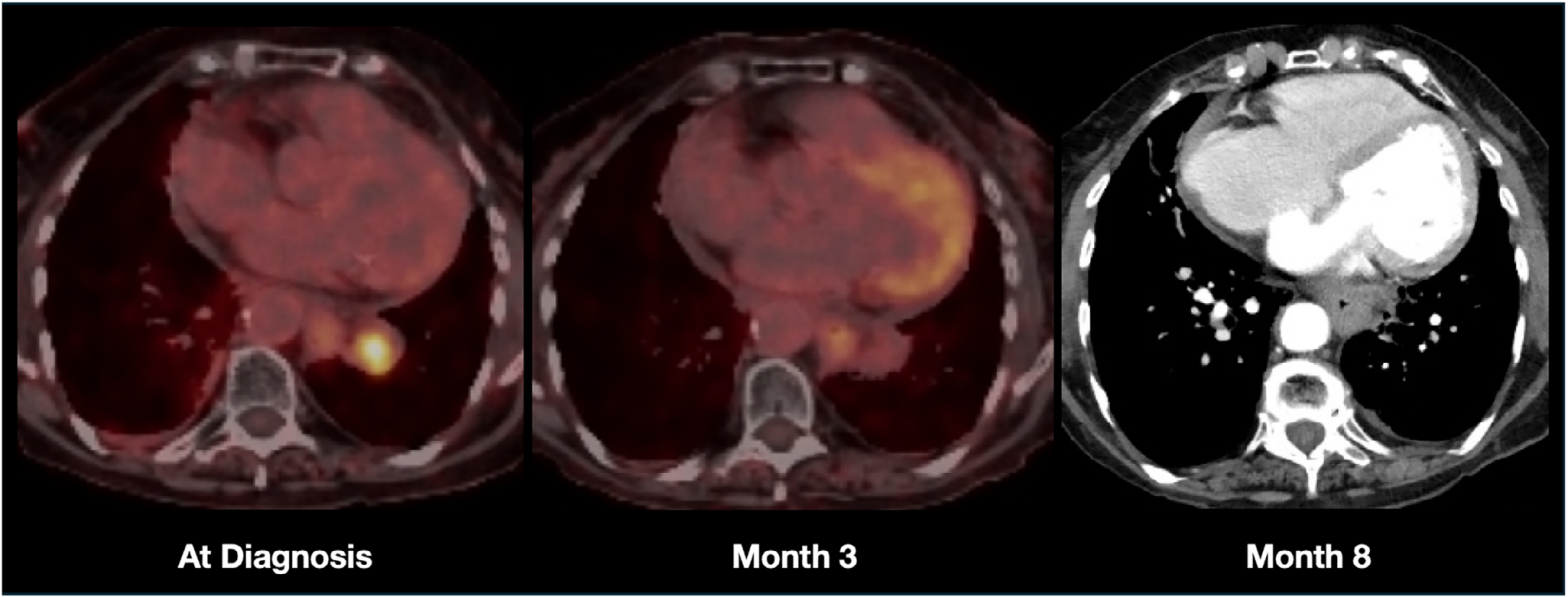
PET/CT at diagnosis and 3 months and CT at 8 months follow‐up of a 78‐year‐old female with metastatic non‐small cell lung cancer (large cell subtype) treated for a centrally located oligometastasis using the Synchrony LWR technique (tumor diameter: 28 mm; GTV: 6.4 cc; PTV: 14.6 cc; 56 Gy/8 fractions).

Another representative case involved a 78‐year‐old female patient with metastatic non‐small cell lung cancer (large cell subtype), treated for centrally located oligometastasis using the Synchrony LWR technique (Figure 2). Treatment planning was performed using MVBH‐CT images. The tumor measured 28 mm in diameter, with a GTV of 6.4 cc and a PTV of 14.6 cc. A total dose of 56 Gy was prescribed in eight consecutive fractions. Complete metabolic tumor response was detected on the 3‐month PET‐CT, and radiological tumor response was confirmed on the 8‐month follow‐up CT.

## 4 DISCUSSION

This study reports the initial clinical experience with the first 20 patients treated for lung tumors using the Radixact Synchrony LWR technique at our institution. When patient suitability was appropriately evaluated during the Synchrony simulation phase, treatments were completed within clinically acceptable durations. Patient‐specific dosimetric verification demonstrated high delivery accuracy. Early clinical outcomes in terms of local control and treatment‐related toxicity suggest that this technique is both feasible and potentially effective.

### 4.1 Patient selection and respiratory stability

Our initial clinical experience with the first 20 patients underscores the critical role of appropriate patient selection for successful implementation of the Synchrony LWR technique. Patients with stable respiratory patterns and well‐defined, radiographically visible targets were more likely to achieve accurate and uninterrupted tumor tracking. Consistent with previous reports, Chen and Ferris demonstrated that the Synchrony system is capable of effectively compensating for phase shifts in patients with regular breathing patterns, whereas Tse et al. identified reduced modeling accuracy in cases with irregular respiration.^20^, ^29^, ^30^ In one of our cases, the patient's breathing pattern became erratic after falling asleep during treatment; however, real‐time audio coaching successfully re‐established respiratory stability, allowing treatment to continue without interruption.

Although Chen et al. recommended a minimum lesion diameter of 20 mm for reliable tracking, our experience demonstrated that the system can successfully manage island‐type tumors as small as 10 mm.^17^ This is further supported by a later report from the same group, in which an 11 mm lesion was effectively treated using the Synchrony LWR technique.^19^

### 4.2 Anatomical and respiratory factors affecting respiratory model reliability

Accurate target localization in the Synchrony LWR technique depends on the alignment of kV radiographs with DRRs generated from the planning CT. Significant anatomical changes between simulation and treatment can compromise this alignment, hindering the generation of a valid respiratory model. To reduce this risk, treatment was typically initiated within 1 to 2 days of CT acquisition. Nevertheless, in one case, a 2‐week delay resulted in pleural effusion and anatomical distortion. A mismatch between the pre‐treatment kVCT and the original planning CT prevented the system from locking onto the TTV, necessitating a new CT scan and replanning.

A similar adaptive intervention was required in a patient with oligometastatic colon adenocarcinoma. Rapid tumor regression observed during the third fraction disrupted tracking stability, requiring a new CT acquisition and TTV redefinition to maintain accurate motion tracking.

In our cohort, all patients deemed suitable for Synchrony‐based treatment during pretreatment evaluation simulation were able to complete therapy using this technique, supporting the reliability of simulation‐based patient selection. The TTV, delineated using the soft tissue window, was typically smaller than the GTV. Since the system tracks the TTV rather than the GTV, density evaluations were based on the TTV. Although TTV density was not prospectively quantified, retrospective review revealed a minimum density of –420 HU, aligning with Kong et al.’s findings that tracking remained accurate in lesions with densities as low as –720 HU (0.280 g/cm^3^).^31^

Among the patients evaluated during simulation, two were excluded from Synchrony treatment due to unsuccessful respiratory model generation. Model failure was attributed to irregular breathing patterns in one patient, and to tumor proximity to the diaphragm and the presence of tumors lacking distinct anatomical demarcation in the other. These patients were subsequently treated using an ITV‐based approach.

### 4.3 Performance evaluation of the Radixact Synchrony LWR system

Unlike conventional systems that rely solely on external surface motion as a surrogate for internal target movement, the Synchrony LWR technique integrates real‐time external signals with internal tumor positions acquired via kV radiographs to build and continuously update a respiratory model.^32^, ^33^, ^34^ This enables accurate adaptation to intrafractional motion.

Adapted from the CyberKnife platform, the Radixact system allows kV radiograph acquisition from up to six patient‐specific gantry angles, enhancing tracking flexibility, particularly in anatomically challenging cases. Yang et al. reported that this capability provided superior modeling accuracy compared to CyberKnife under suboptimal conditions.^35^ In addition to imaging flexibility, the system's ability to perform accurate motion‐adapted delivery is further supported by its low latency characteristics. The potential impact of total latency in the Radixact system on real‐time motion tracking accuracy has been previously characterized by Schnarr et al., with latency values reported for both MLC (70 ms) and jaw (30 ms) corrections, supporting the feasibility of precise motion‐adapted dose delivery.^36^

In our study, treatment durations exceeded beam‐on times primarily due to model rebuilding during delivery. Although intrafractional interruptions occurred, no treatments were discontinued, and the interruption rate was consistent with prior reports.^17^, ^20^, ^34^ Factors contributing to interruptions included irregular breathing, diaphragm–tumor phase shifts, and conservative tracking thresholds. Setting a short auto‐pause window (e.g., 10 s) may improve modeling accuracy but increases treatment time.^20^, ^34^

As extended treatment times may affect patient comfort and compliance, reduced cooperation could further increase the likelihood of interruptions. Therefore, selecting patients with good performance status and the ability to remain cooperative throughout the session is essential. For patients who are unlikely to tolerate prolonged sessions, an alternative motion management strategy such as the ITV‐based approach may be preferable to facilitate smoother treatment delivery. Notably, previous studies have shown that the Synchrony system can maintain acceptable dosimetric accuracy even in the presence of such interruptions.^20^

DIBH and gating techniques are also known to prolong treatment. For example, Oh et al. observed treatment time increases of up to 200% depending on respiratory stability.^37^ Fuji et al. noted that tighter gating thresholds reduced residual motion but extended treatment duration significantly.^38^

The mean radiographic imaging dose per fraction was 0.90 cGy, consistent with previously reported data, and this value is significantly lower than typical cone‐beam CT doses.^30^ Although reducing radiograph numbers can lower dose, it may compromise model accuracy.^39^ In our cohort, kV imaging contributed only 0.11% of the prescribed dose, lower than the 0.5% reported by Chen et al., possibly due to smaller GTVs or different planning parameters.^17^

Both gantry rotation and couch translation during fluence delivery introduce time‐dependent characteristics to helical radiotherapy, making it particularly susceptible to interplay effects. This concern is especially critical when respiratory‐induced tumor motion occurs along the superior‐inferior (SI) axis, which coincides with the direction of couch translation and may interfere with the sequential “slice‐by‐slice” dose deposition.^40^ Such motion‐related interference can lead to deviations between the planned and delivered dose distributions, particularly in thoracic tumors with large motion amplitudes.^41^ Nevertheless, the findings by Kanagi et al. support the clinical feasibility of helical tomotherapy for lung SBRT when standard margin expansions are applied.^42^

Moreover, various phantom‐based dosimetric studies simulating respiratory motion have demonstrated that the Radixact Synchrony system can achieve dose delivery accuracy comparable to that of static targets.^21^ It has also been shown that baseline shifts of up to 3 mm per min and amplitude variations of up to 40% have negligible impact on tracking performance and dose accuracy.^43^ These findings indicate that the system can maintain robust tracking performance even under moderate respiratory variability, supporting its clinical reliability for accurate dose delivery in thoracic radiotherapy.

Consistent with these findings, our patient‐specific QA results also confirmed the high dosimetric precision of the Synchrony LWR system. Both gamma analysis and absolute point dose measurements demonstrated excellent agreement between planned and delivered doses under motion, confirming that the Synchrony LWR system achieves high dosimetric accuracy comparable to static delivery.^30^, ^44^, ^45^, ^46^

### 4.4 Early clinical outcomes

SBRT has demonstrated high rates of local control in both primary and secondary lung malignancies.^47^, ^48^, ^49^, ^50^ However, inadequate management of respiratory‐induced tumor motion during treatment delivery has been associated with compromised local control.^51^, ^52^ Additionally, failure to compensate for intrafractional motion may increase the likelihood of radiation‐induced toxicities, particularly in peripheral lesions near the chest wall and ribs, as well as in centrally located tumors adjacent to critical structures such as major blood vessels, central airways, and other mediastinal organs.^53^, ^54^, ^55^, ^56^

In our patient cohort treated with the Synchrony LWR technique, high local control rates were achieved without any significant acute or late toxicities. These outcomes suggest that the Synchrony LWR technique may offer a precise and effective approach for managing respiratory‐induced tumor motion in lung SBRT.

### 4.5 Limitations of the study

This study was conducted with a limited number of patients at a single center, which may restrict the generalizability of the findings. Future studies involving a larger patient cohort may help improve the generalizability and robustness of the findings. Additionally, due to the retrospective nature of data collection, the causes of respiratory model failures could not be quantitatively assessed. Moreover, long‐term clinical outcomes such as local control, toxicity, and overall survival were not evaluated in this study. Therefore, the impact of this technique on patient outcomes could only be inferred indirectly.

## 5 CONCLUSION

The Radixact Synchrony LWR system offers an effective, non‐invasive solution for respiratory motion management in lung SBRT, achieving clinically acceptable treatment durations. Our early clinical results indicate favorable local control and low toxicity, supporting its feasibility in well‐selected patients. Further studies with larger cohorts and extended follow‐up are warranted to validate and generalize these initial findings.

## AUTHOR CONTRIBUTIONS

**Evren Ozan Goksel**: Conceptualization; data curation; formal analysis; methodology; writing—originial draft. **Zeynep Ozen**: Data curation. **Seckin Gunduz**: Data curation. **Artunc Ture**: Data curation. **Halil Kucucuk**: Data curation. **Alptekin Arifoglu**: Data curation. **Evrim Tezcanli**: Data curation; writing— review and editing. **Ufuk Abacioglu**: Conceptualization, data curation, methodology, writing—review and editing. **Meric Sengoz**: Writing—review and editing.

## CONFLICT OF INTEREST STATEMENT

Evren Ozan Goksel and Ufuk Abacioglu have received speaker honoraria from Accuray Inc. The authors have no other relevant affiliations or financial involvement with any organization or entity with a financial interest in or financial conflict with the subject matter or materials discussed in the manuscript. No writing assistance was utilized in the production of this manuscript.

## ETHICS STATEMENT

The authors state that they have obtained appropriate institutional review board approval or have followed the principles outlined in the Declaration of Helsinki for all human or animal experimental investigations. This study was deemed ethically appropriate by the Acibadem Mehmet Ali Aydinlar University Ethics Committee, with the reference number 2024–15/613.

## Data Availability

The datasets generated during and/or analyzed during the current study are available from the corresponding author on reasonable request.
